# Indoor microbial exposure increases complement component C3a and *C*-reactive protein concentrations in serum

**DOI:** 10.1016/j.heliyon.2024.e24104

**Published:** 2024-01-08

**Authors:** Outi Karhuvaara, Liisa Vilén, Jari Nuutila, Tuula Putus, Janne Atosuo

**Affiliations:** a The Laboratory of Immunochemistry, Department of Biotechnology, Faculty of Science and Engineering, University of Turku, Turku, Finland; b Environmental Medicine and Occupational Health, Department of Clinical Medicine, Faculty of Medicine, University of Turku, Turku, Finland

**Keywords:** Moisture damage, Complement, Anaphylatoxins, *C*-reactive protein, Inflammation

## Abstract

Indoor exposure to microbial growth, caused by moisture damage, has been an established health risk for several decades. It is likely that a damp indoor environment contains biological pollutants that trigger both the innate and adaptive branches of the immune system.

In this study, we investigated the association between moisture damage related microbial exposure and serum C3a, C5a and CRP concentrations in Finnish adults.

Serum C3a and CRP concentrations were elevated in individuals exposed to moisture damage and microbial growth in an indoor air environment. The elevated concentrations may be due to environmental factors present in moisture-damaged buildings. Complement activation and the resulting proinflammatory cleavage products may be a driving factor in inflammatory responses following exposure to indoor moisture damage and related microbial growth.

## Editor highlights

• Individuals with long term exposure indoor mold exhibit elevated concentrations of *C*-reactive protein.
• Complement activation product C3a concentrations are higher on average in exposed individuals.
• Findings corroborate previous observations, where indoor mold exposure was found to activate complement classical pathway.

## Abbreviations

IgG: Immunoglobulin G
CRP: C-reactive protein
C3a: complement component 3a
C5a: complement component 5a
IL-6: Interleukin 6
TNF-α: Tumor Necrosis Factor α
IL-1β: Interleukin 1β
IC: Immune complex
OD: Optical density
ELISA: Enzyme linked immunosorbent assay

## 1 Introduction

Poor indoor air quality due to moisture damage and associated microbial growth is a common worldwide problem. In Finland, a significant percentage of the working population is annually exposed to moisture damage related microbes and other contaminants.

Correlation between moisture damage and adverse health effects has been established in several studies over a number of years [[1], [2], [3], [4], [5], [6], [7], [8], [9], [10], [11], [12], [13], [14]], yet the underlying mechanism causing the symptoms remains unclear. Assessing the potential causal factors as well as the immunological pathways leading to exposure-associated symptoms remains a challenge. Previous studies have shown that several humoral components of the immune system may be relevant in the development of symptoms following prolonged exposure to moisture damage and related microbial growth. Exposed individuals exhibit higher concentrations of microbe spore specific immunoglobulin G (IgG) in their serum [15]. Exposed individuals also exhibit higher complement activity [16]. Studies conducted on cell cultures and animal models have shown that exposure to mold particles induces proinflammatory responses [[17], [18]]. Mold particles have been shown to induce the production of proinflammatory cytokine in bronchial epithelial cells and macrophages [[19], [20], [21], [22], [23]].

The humoral components of the innate immune system consist mainly of the complement system and pattern recognition molecules, such as pentraxins [24]. These molecules are crucial in removing both foreign and endogenous unwanted particles, and disturbances in their homeostasis have a negative impact on the host's health.

*C*-reactive protein (CRP), a commonly marker for inflammation and clinical infections [25,26], interacts with the complement system and augments its effects [27]. Its expression is induced by proinflammatory cytokines, mainly IL-6, Il-1β and TNF-α [26]. Normal circulating concentrations of CRP are below 1 mg/l in healthy adults. Concentrations exceeding 10 mg/l are typically considered a sign of a clinical infection or an inflammatory state, but slightly elevated concentrations below 10 mg/l have been shown to correlate with low-grade systemic inflammation and various health risks [[28], [29], [30]].

Serum CRP has a protective role against bacterial infections, principally through the activation of complement system and the subsequent opsonization of pathogens [31,32]. CRP is a known activator of the classical pathway, and is most effective during the early steps of activation. Conversely, CRP inhibits the alternative pathway by decreasing C3 and C5 convertase activities, most likely in order to prevent excessive damage by the complement system [31]. CRP also initiates cell-mediated immune responses by binding to the Fc receptors of IgG [31,32].

All complement activation pathways lead to the cleavage of C3 and C5, generating proinflammatory fragments C3a and C5a respectively, which are among the most powerful chemotactic agents released during complement activation. They are crucial in activating innate immune responses such as phagocytosis, oxidative burst, degranulation, and cytokine production against pathogens and other danger-associated molecules [33,34,35,[36], [37], [38], [39]], but also a contributing factor in adaptive immune responses [40,[41], [42], [43], [44], [45]]. A previous small-scale report found elevated concentrations of C3a and C4a in the tear fluid of individuals exposed to indoor mold [46].

The complement system, its components and modulators are potential biomarkers for indoor mold exposure and the accompanying symptoms. The objective of this study was to investigate the relationship between indoor microbial exposure and C3a, C5a and CRP. We measured the serum concentrations of C3a, C5a and CRP in Finnish adults working or living in buildings with verified moisture damage and microbial growth.

## 2 Materials and methods

### 2.1 Participants and buildings

The study was conducted with the participation of adults in Southern and Southwestern Finland working in public buildings and residing in private apartment buildings. All buildings were chosen so that they represent typical Finnish building stock in the branch, i.e. health care centers, schools, fire stations and office buildings. Additionally, they had to be inspected by local building authorities, health inspection and microbiological samples had been taken prior to renovation.

All building users were invited from each building irrespective to the fact that they had symptoms or not. Similarly, reference buildings were selected so that they were built in the same era, the use of the building was the same. All building users in the reference building were also invited to participate the study.

The exposed group consisted of adults working or living in moisture-damaged buildings with verified microbial growth. The reference group was not exposed to moisture damage or related microbial growth in the reference building. The total number of participants was 237 (152 exposed, 85 reference, Table 1).

**Table 1:** The observed microbial damage of the buildings and the numbers of paricipants from each building.

	Number of Paricipants	Microbial Damage (±)
Damaged buildings, total	152	
Health care center	35	+
Fire Station 1	26	+
Apartment building	18	+
High School	43	+
Secondary School	32	+
Reference buildings, total	88	
Health care center	58	–
Fire station 2	11	–
Fire station 3	12	–
Fire station 4	7	–

### 2.2 Ethical considerations

This study was performed according to the clinical standards of the Declaration of Helsinki. The study plan was approved by the ethical committee of Turku University Central Hospital (Dnro: 59/1801/2019), and written informed consent was obtained from the participants. The data was handled according to General Data Protection Regulations (GDPR).

### 2.3 Evaluation of moisture damage and microbial growth in buildings

Indoor dampness as well as structural damage was evaluated and microbial samples collected by independent third parties. Microbe samples were analyzed utilizing the microbe cultivation methods accredited by the Finnish Accreditation Service (FINAS). The moisture-damage microbial panel contained the following indicator microbes: *Aspergillus versicolor, Chaetomium globosum, Fusarium merismoides, Stachybotrys chartarum, Streptomyces albus, Streptomyces halstedii, Trichoderma citrinoviride* and *Tritirachium oryzae*. Buildings containing growth of at least three moisture indicator species in addition to excessive indoor moisture and structural damage were classified as damaged, while the reference buildings contained no observable moisture damage or microbial growth.

All samples collected from buildings were analyzed utilizing the methods accredited by the FINAS. The cultivation process was done with a dilution culture. The total bacterial content and the actinobacterial content were determined in THG medium (tryptone-yeast extract -glucose – agar), the total spore content of mesophilic fungi in MA2 medium (Malt – agar 2 %), and the total spore content of xerophilic fungi in DG-18 medium (dichloran-glyserol – agar). The target buildings and the number of participants from each building are presented in Table 1. The refence buildings lacked the damage indicator microbes. These indicator species are internationally defined, and this group of microbes contains the fungi and bacteria commonly found at damaged sites [47].

### 2.4 Serum samples

One blood sample was collected from the participants during their workday in the target building. Samples were collected in 8-ml Vacuette serum tubes (with gel) (Greiner Bio-one, Kremsmünster, Austria). The serum samples were prepared by centrifuging the blood samples at 1800 *g* for 10 min and then distributing the serum into 2-ml Eppendorf tubes and storing at −80 °C.

### 2.5 Serological analyses

The C3a and C5a concentrations were measured with enzyme linked immunosorbent assay (ELISA) using commercially available kits (Elabscience, Houston, Texas, USA). Proper serum concentrations for the analyses were determined with serum samples from healthy volunteers. Assays were performed according to the manufacturer's instructions using 2 % dilution of the serum samples. The OD 450 nm was measured with a plate reader (Hidex, Turku, Finland).

Serum CRP concentrations were measured turbidometrically with Quickread Go (Orion, Finland). The instrument was a point of care method with a detection limit of 1 mg/l of CRP in the serum.

### 2.6 Data analysis

Raw data was stored in. xls format. Data was analyzed with Excel and Origin (Microcal, OriginLab, Massachusetts, USA). The C3a and C5a concentrations were determined by plotting a four parameter standard curve and calculating the concentrations based on the curve, as instructed by the kit manufacturer.

Statistical analysis was performed with IBM SPSS Statistics version 26 (IBM, New York, USA). Differences between groups were considered significant at p < 0.05. C3a concentrations were normally distributed, and Student's t-test was used compare the means between the groups. The Mann-Whitney *U* test was used for C5a due to the non-normal distribution. The incidence of outliers was analyzed by transforming C5a concentrations into categorical variables where the outliers were defined as the 75th percentile +1.5* interquartile range. The differences in the portion of outliers was analyzed with Pearson's chi-square.

Individuals in the exposed and reference groups were distributed into three categorical subgroups according to their CRP-concentrations: <1 mg/l, 1–10 mg/l and >10 mg/l. Differences in the subgroup distributions between the exposed and reference groups were analyzed with Pearson's chi-square test.

## 3 Results

Age, sex and immunological marker distributions are presented in Table 2. The values for C3a serum concentrations were normally distributed (Shapiro-Wilk's test of normality p = 0.648), while the distribution for C5a concentrations was nonparametric (Shapiro-Wilk's test of normality p < 0.001).

**Table 2:** **Descriptive statistics and statistical tests of the exposed and reference group**. The p-value for C3a concentrations was calculated with Student's t-test. C5a concentrations were nonparametrically distributed and statistical significance was calculated with Mann-Whitney *U* test. The statistical significance of C5a level outliers was calculated with Pearson's chi square test. The differences in frequencies of CRP level categories were calculated with Pearson's chi square test.

	Exposed	Reference	
Age (years) Sex (% female)	47 ± 1 65.8	43 ± 1 60.0	
C3a			
Mean ± SE CI 95 % Lower Bound Upper bound Median	221 ± 5.2 210 230 213	181 ± 5.8 169 191 177	Student's T-test p < 0.001^a^
C5a			
Mean ± SE CI 95 % Lower Bound Upper bound Median <40.1 Outliers (≤40.1 ng/ml)	21.7 ± 1.0 19.8 23.6 17.9 139 13	21.0 ± 0.9 19.0 22.6 19.4 86 2	Mann-Whitney *U* test p = 0.475 χ2 (2) = 3.751 p = 0.053
CRP			
<1 mg/l 1–10 mg/l >10 mg l	85 60 7	63 24 1	χ2 (2) = 6.602 p = 0.031^a^

a = Statistically significant.

**Fig. 1:**
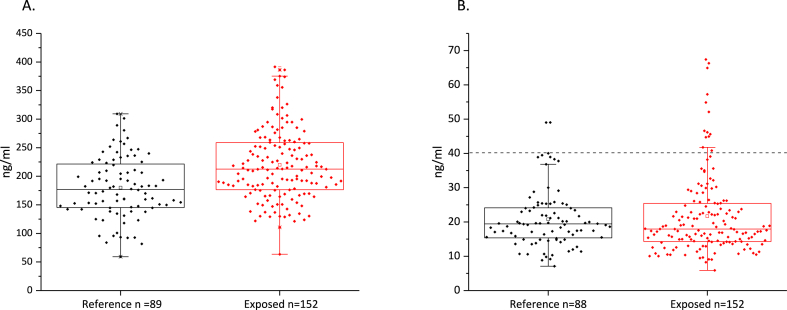
A. Boxplot of serum C3a levels in reference group and exposed group. B. **Boxplot of serum C5a levels in reference group and exposed group.** The cutoff line for outliers is 40.1 ng/ml 8.2 % of the exposed group were outliers versus 2.3 % in the reference group.

The distributions are represented in Fig. 1A and B. The group exposed to moisture damage had, on average, higher concentrations of serum C3a, and the difference was statistically significant (p < 0.001, Table 2). Although there was no statistically significant difference in the mean C5a concentrations between the groups (Table 2), the exposed group exhibited more outliers (8.2 %) compared to the reference group (2.3 %) (Fig. 1B). The chi square test p-value was 0.053, thus the difference in outlier percentages between the groups was not quite statistically significant.

CRP was measured with a point-of-care method with the detection limit of 1 mg/l, and the majority of samples had a concentration below the detection limit. Therefore, the distribution of CRP could not be accurately determined, and a categorical statistical method was used instead. Most of the samples in both groups had a serum CRP concentration <1 mg/l (Table 2). Concentrations of 1–10 mg/l were considered subclinical values, while concentrations over 10 mg/l were considered indicative of a clinical infection or inflammation. The exposed group had higher incidences of both subclinical and clinical elevations of CRP concentrations (Table 2), and the difference was statistically significant (p = 0.031).

## 4 Discussion

Estimating the duration and severity of an individual's exposure to poor indoor air remains difficult, as progressively worsening symptoms are initially unclear and often almost imperceptible. In addition, the microbiome of moisture-damaged buildings can vary widely from case to case. In this study, the condition of the buildings was investigated with assessment of structural damage as well as with microbiological cultivation methods; the building was determined to be a damaged if three out of eight internationally defined indicator microbes were found in the collected samples, in addition to structural damage and dampness.

It is possible that the microbes themselves are not the sole causative agent behind the indoor air related symptoms, as a moisture damaged indoor environment contains multiple potentially harmful components. The numerous potentially irritating or even toxic compounds (cell wall components, spores, mycelia, hyphae, toxins) they produce and release are nonetheless a very likely contributing factor. It is worth noting that the majority of the immune responses among the exposed individuals are localized in the airways and lungs; this is where persistent and prolonged contact of microbial particles with the host immune system occurs. These localized responses remain difficult to measure with non-invasive methods, such as serological analyses.

While typical moisture damage related microbes are rarely pathogenic to non-immunocompromised individuals, they may nevertheless stimulate both innate and adaptive inflammatory responses. We have previously shown that exposure to moisture damage related microbes stimulates the production of spore (from *Streptomyces albus* and *Aspergillus versicolor*) specific antibodies, specifically IgG1 and IgG3 [15], which are major activators of the complement classical pathway. Correspondingly, elevated classical pathway activity has been observed in exposed individuals in our previous study [16].

CRP is one of the most established and widely used nonspecific markers for inflammation and infection. CRP expression is induced by proinflammatory cytokines such as IL-6, the production of which can be induced by mold particles [[19], [20], [21], [22], [23],^48^,^49^]. In clinical studies, observation of the effect of microbe exposure on cytokine production has been a challenge due to inter-individual differences and the rather transient nature of cytokine production. The higher incidence of elevated CRP concentrations following mold exposure in this study indicates that CRP could be a marker for the proinflammatory responses caused by indoor mold. More sensitive analyses are required in the future to measure the quantitative effects of exposure.

While the role of CRP remains somewhat incompletely understood, its role in modulating the effects of the complement pathway is relatively well studied. CRP is an activator of the early stages of the complement classical pathway, leading to the cleavage of C3a. Conversely, it induces very little activation of C5 convertase and the subsequent cleavage of C5a, most likely in order to limit the strong and potentially harmful inflammatory effects [31,32].

Complement activation leads to the release of several activation fragments, the most prominent being C3a, C4a and C5a. C3a and C5a were selected for this study because they are the most researched and potent proinflammatory complement cleavage products. Our primary interest was whether these proinflammatory molecules are present in elevated quantities in exposed individuals.

The average C3a concentrations were higher in the exposed group than the reference group, and the difference was statistically significant. The elevated concentrations of C3a observed in this study therefore strengthen the hypothesis that increased antibody and CRP production lead to the activation of the complement classical pathway.

Average serum C5a concentrations did not differ between the groups, although the exposed group had a higher percentage of outliers. It is possible that elevated CRP upregulates the early steps of the complement pathway, while inhibiting the later steps, including the cleavage of C5a. Moderate complement activation triggered by mold exposure may thus not result in statistically significant differences between the groups.

These findings show that there is a correlation between indoor mold exposure, C3a and CRP. All of these molecules are instrumental in humoral immune responses against foreign bodies and pathogens, and they play an important role in the interplay between the innate and adaptive immune systems. Elevated C3a and CRP concentrations in the exposed group are in line with our previous findings with the same study participants; these findings showed that the elevated microbe-specific antibody concentrations of the exposed group correlated with the increased activity of the classical complement pathway. The potential causal relationship between elevated concentrations of microbe-specific antibodies, increased complement activity, elevated C3a and CRP concentrations and, finally, vague inflammatory symptoms in adults working or living in moisture damaged buildings needs to be investigated further. More research is needed on both the potential causative factors in the indoor environment as well as the defense reactions of the host.

### 4.1 Limitations of the study

The major limitation of this study is the assessment of indoor microbial exposure outside the investigated target building for each individual participant. Exposure outside the target building could not be assessed with the resources available for this study. Without microbiological analyses, questionnaires are also not a reliable measure for exposure assessment, as microbial growth cannot be verified as the source of indoor air problems. However, the study participants’ duration exposure is significant compared to the reference group, and meaningful conclusions and discussion can be drawn from this study design.

CRP is a nonspecific marker for inflammation, and there exist alternative causes for elevated concentrations in serum. It cannot be individually used as evidence of the causative link between exposure and health effects, but must be considered together with other markers and clinical findings. Additionally, CRP levels were measured using a relatively insensitive point-of-care method, and more sensitive methods should be implemented to achieve a better understanding on the relationship between indoor microbial exposure and CRP. Nonetheless, the results of this study show a show a clear association between the two.

Complement components are also unspecific inflammation markers, and serum levels of anaphylatoxins are not very well defined. Differing concentrations could be caused by underlying diseases and phenotypical differences in addition to environmental factors, such as indoor microbial exposure. The polymorphic nature of complement component proteins could contribute to the differing rates of C3 cleavage and therefore concentrations of C3a in serum [50]. Difference in C3 phenotypes could not be investigated, therefore its effect on observed differences between groups could not be evaluated in this study. On the other hand, polymorphism could also be a factor in the development of symptoms and disease following exposure to moisture damage related microbes. The role of complement component polymorphism is an interesting subject of investigation for future research on this subject.

## 6 Conclusions

Exposure to indoor mold is associated with elevated CRP and C3a concentrations in serum, while the effect on C5a concentrations is ambiguous. This study, along with our previous studies, suggests that the continuous presence of microbial antigens, caused by prolonged exposure to indoor mold, is a potential contributing factor behind increased complement system activity. Moreover, the proinflammatory cleavage products of the complement cascade further mediate the inflammatory responses, contributing to the symptoms and diseases commonly associated with indoor air dampness and microbial growth. Further research on subject should focus on the inflammatory effects of complement system activation and the antibody-antigen complexes that activate the complement cascade.

## Data availability statement

Data used in the study are available upon reasonable request. Date are stored in a controlled access storage at University of Turku.

## Additional information

No additional information is available for this paper.

## Funding

This work was funded by the Finnish Work Environment Fund (Finland) and by the Parliament of Finland.

## Ethics

This study was performed according to the clinical standards of the Declaration of Helsinki. The study plan was approved by the ethical committee of the Turku University Central Hospital (Dnro: 59/1801/2019), and a written informed consent was obtained from every building user who participated.

## CRediT authorship contribution statement

**Outi Karhuvaara:** Data curation, Formal analysis, Investigation, Methodology, Visualization, Writing – original draft, Writing – review & editing. **Liisa Vilén:** Investigation. **Jari Nuutila:** Conceptualization, Supervision, Writing – review & editing. **Tuula Putus:** Conceptualization, Funding acquisition, Project administration, Resources, Supervision. **Janne Atosuo:** Conceptualization, Funding acquisition, Investigation, Methodology, Project administration, Supervision, Writing – review & editing.

## Declaration of competing interest

The authors declare that they have no known competing financial interests or personal relationships that could have appeared to influence the work reported in this paper.
